# Nogo receptor impairs the clearance of fibril amyloid‐β by microglia and accelerates Alzheimer’s‐like disease progression

**DOI:** 10.1111/acel.13515

**Published:** 2021-11-24

**Authors:** Jianing Wang, Xiaoying Qin, Hao Sun, Meijun He, Qunyu Lv, Congcong Gao, Xinran He, Hong Liao

**Affiliations:** ^1^ New drug screening center, Jiangsu Center for Pharmacodynamics Research and Evaluation China Pharmaceutical University 24 Tongjiaxiang Street Nanjing 210009 China

**Keywords:** alzheimer's disease, Aβ, microglia, NgR, phagocytosis

## Abstract

Alzheimer's disease (AD) is characterized by the progressive accumulation of β‐amyloid (Aβ)‐containing amyloid plaques, and microglia play a critical role in mediating Aβ clearance. Mounting evidence has confirmed that the ability of microglia in clearing Aβ decreased with aging and AD progress, but the underlying mechanisms are unclear. Previously, we have demonstrated that Nogo receptor (NgR), a receptor for three axon growth inhibitors associated with myelin, can decrease adhesion and migration of microglia to fibrils Aβ with aging. However, whether NgR expressed on microglia affect microglia phagocytosis of fibrils Aβ with aging remains unclear. Here, we found that aged but not young microglia showed increased NgR expression and decreased Aβ phagocytosis in APP/PS1 transgenic mice. NgR knockdown APP/PS1 mice showed simultaneous reduced amyloid burden and improved spatial learning and memory, which were associated with increased Aβ clearance. Importantly, Nogo‐P4, an agonist of NgR, enhanced the protein level of p‐Smad2/3, leading to a significant transcriptional inhibition of CD36 gene expression, which in turn decreased the microglial phagocytosis of Aβ. Moreover, ROCK accounted for Nogo‐P4‐induced activation of Smad2/3 signaling. Finally, the decreasing effect of NgR on microglial Aβ uptake was confirmed in a mouse model of intra‐hippocampal fAβ injection. Our findings suggest that NgR may play an important role in the regulation of Aβ homeostasis, and has potential as a therapeutic target for AD.

## Graphical abstract

Schematic diagram of the effects of NgR on microglial of Aβ phagocytosis. NgR decreases Aβ phagocytosis through ROCK‐Smad2/3‐CD36 signaling, but not the NF‐кB or PPARг pathway.

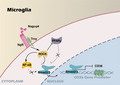

## 1 INTRODUCTION

Alzheimer's disease (AD) is the most common form of dementia in the elderly (Tanzi, 2012). Mounting evidence suggests that the accumulation and aggregation of amyloid‐β (Aβ) are the key initiating event in the pathogenesis of AD (Hardy & Selkoe, 2002). Failure of Aβ clearance or disruption of Aβ homeostasis results in an accumulation of Aβ in the brain. Late‐onset AD patients exhibit compromised Aβ clearance rather than overproduction of Aβ; thus, impairment of Aβ clearance is a common antecedent to late‐onset AD (Mawuenyega and Sigurdson et al., 2010).

Microglia are immune cells that contribute to phagocytosis in the central nervous system, and play critical roles in the uptake of Aβ and dead neural cells (Mandrekar‐Colucci & Karlo, 2012). There is an age‐dependent decrease in the ability of microglia to phagocytose Aβ fibrils, as well as a decrease in the expression of Aβ‐interacting protein *in vitro* (Floden & Combs, 2011). Thus, microglia are emerging as critical regulators of innate immune responses in AD, and understanding the molecular and cellular mechanisms that cause microglial dysfunction may lead to the identification of strategies to restore healthy microglial function and prevent the development of AD.

The Nogo receptor (NgR), as a receptor for three axon growth inhibitors associated with myelin, has been extensively studied due to its role in triggering growth cone collapse and arresting neurite/axon growth (Fournier & GrandPre, 2001). In addition, recent studies have provided direct evidence of a role of the NgR in AD. For example, in patients with AD and elderly rats with spatial cognitive deficits, Nogo‐A and NgR expression are increased (Gil & Nicolas, 2006; VanGuilder & Sonntag, 2013; Zhu & Guo, 2007), suggesting their potential roles in AD pathogenesis and progression. Furthermore, NgR physically interacts with amyloid precursor protein (APP) and Aβ in a pocket on the concave surface, which can be distinguished from the surface required for Nogo‐66 binding (Park & Gimbel, 2006). Subcutaneous NgR (310) ecto‐Fc treatment reduces the brain Aβ plaque load and improves spatial memory in APPswe/PSEN‐1ΔE9 transgenic mice (Park & Widi, 2006). Under normal circumstances, NgR is reported to be detected mainly in neurons (Zhang et al., 2014), but in some pathologic state including AD, NgR is also expressed on the surface of microglia and has indispensable effects (Fang & Yao, 2016; Satoh et al., 2005). Our previous study demonstrated that the Nogo/NgR pathway plays a role in the Aβ pathology that characterizes AD by modulating microglial adhesion and migration to Aβ (Fang & Wang, 2018). However, the effects of NgR expressed on microglia in Aβ phagocytosis and the underlying molecular mechanism are not entirely understood.

Cluster of differentiation 36 (CD36) is a member of the class B scavenger receptor family, which could transport various molecules into cells such as fatty acids, collagen, and oxidized low‐density lipoproteins (Endemann & Stanton, 1993). CD36 as a major pattern recognition receptor expressed on microglia had been show to bind to Aβ (El & Moore, 2003), and impaired recycling of CD36 reduces the phagocytic efficiency of microglia toward Aβ (Lucin & O'Brien, 2013). Moreover, CD36 is increased by blockade of the Nogo/NgR pathway in the microglia of double transgenic mice expressing a chimeric mouse/human APP and mutant human presenilin 1 protein (APP/PS1) mouse (Fang & Wang, 2018). In addition, Nogo‐66 binding to NgR inhibits the adhesion and migration of microglia through the RhoA/Rho‐associated kinase (ROCK) pathway *in vitro* (Yan & Zhou, 2012). However, the precise mechanisms involved remain unclear. Blockade of transforming growth factor beta (TGF‐β)‐activated Smad2/3 reportedly increases Aβ phagocytosis (Town & Laouar, 2008), and TGF‐β signals through Smad2/3 to downregulate the scavenger receptor CD36 (Rustenhoven & Aalderink, 2016). Thus, microglial NgR appears to influence Aβ phagocytosis through ROCK‐Smad2/3‐CD36 signaling.

Here, we examined the role of NgR in microglial phagocytosis that occurs in AD using microglia of different ages, and found that aging microglia exhibited decreased Aβ clearance in APP/PS1 transgenic mice. Conditional knockdown of NgR reduces amyloid burden and improves learning and memory by enhancing Aβ phagocytosis in the brain. In addition, we elucidated that NgR knockdown promoted Aβ phagocytosis by regulating ROCK‐Smad2/3‐CD36 signaling in primary microglia. Furthermore, NgR decreased microglial Aβ uptake in a mouse model of intra‐hippocampal fAβ injection. Together, NgR signaling mediates an age‐dependent decrease microglial Aβ phagocytosis in the development of AD‐like pathology, suggesting that NgR is a potential therapeutic target for AD.

## 2 RESULTS

### 2.1 Aging decreases Aβ phagocytosis and upregulates NgR levels in APP/PS1 mice microglia

**FIGURE 1:**
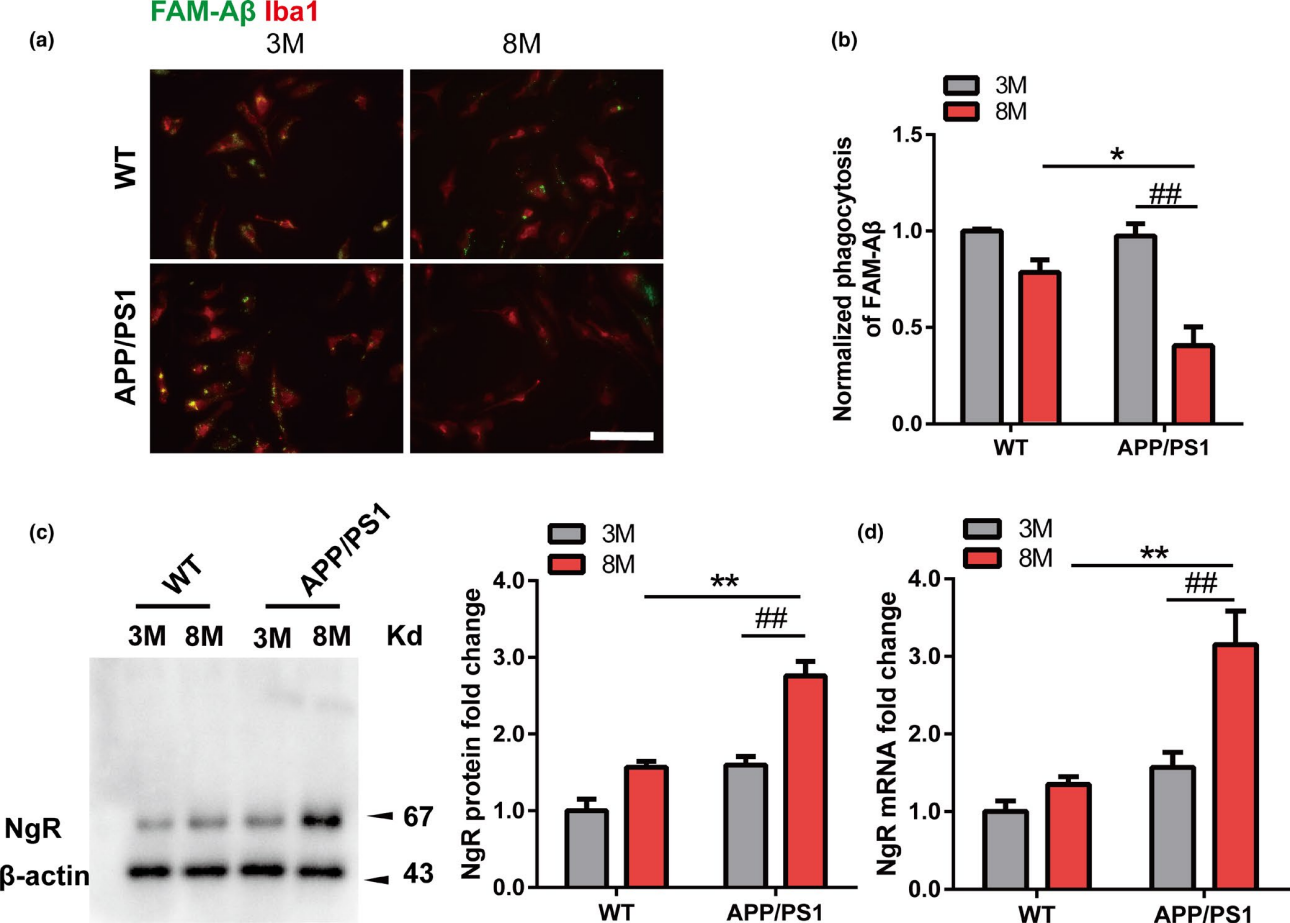
Microglia shows age‐dependent decline in Aβ phagocytosis and NgR upregulation from APP/PS1 mice. (a) Representative images of phagocytosed by primary microglia (Iba‐1, red) isolated from 3‐month‐old or 8‐month‐old WT mice and APP/PS1 mice after incubated with 0.5 μM FAM‐Aβ (Anaspec, green) for 4 h. Scale bar: 20 μm. (b) The quantification of the phagocytosis assay. *n *= 3wells/group, and *N* = 3 independent experiments (data presented are the average of all experiments). (c‐d) NgR protein (c) and mRNA (d) levels was measured in microglia isolated from 3‐month‐old or 8‐month‐old WT mice and APP/PS1 mice. *n* = 3–5 samples/group in every experiment, and *N* = 3 independent experiments. Values were reported as the mean ±SEM. Statistical analyses were performed using a two‐way ANOVA with Bonferroni's multiple‐comparison post hoc test, *p < 0.05, **p < 0.01 for microglia from 8‐month‐old APP/PS1 mice vs. 8‐month‐old WT mice; and #*p* < 0.05, ##*p* < 0.01 for microglia from 8‐month‐old APP/PS1 mice vs. 3‐month‐old APP/PS1 mice

Microglia play a critical role in AD by contributing the clearance of Aβ, but the ability of microglia to clear Aβ may decrease with age in the APP/PS1 mouse (Hickman & Allison, 2008). To further confirm whether aging affects Aβ phagocytosis, primary microglia isolated from APP/PS1 mice and their age‐matched wild‐type (WT) littermates, in both young (3 months) and aged (8 months) mice, were incubated in the presence of green fluorescein amidite (FAM)‐Aβ (0.5 μM). After 4 h exposure to Aβ, intracellular levels of Aβ were determined. The uptake of Aβ was significantly decreased in aged, but not young microglia in APP/PS1 mice and slightly dampened in WT mice (no significant difference) (Figure 1a‐b). Consistent with this, at 8 months of age microglia from APP/PS1 transgenic mice showed significantly reduced Aβ phagocytosis compared to their age‐matched WT littermates, but there was no significant difference at 3 months of age (Figure 1b). Western blotting and quantitative PCR (qPCR) were used to investigate whether aging or AD transgene also induced NgR expression changes in microglia, respectively. Aged microglia expressed a higher NgR expression compared to young microglia from APP/PS1 transgenic mice, and it also can be seen in WT mice that the expression of NgR slightly increased over time (no significant difference) (Figure 1c‐d). Interestingly, at the 8 months, microglial NgR expression was much higher in APP/PS1 transgenic mice than WT mice instead of 3 months, which indicated that AD transgene and aging together induced the increase of NgR expression in microglia. Taken together, these findings suggest that microglia show age‐dependent upregulation of NgR in the APP/PS1 mouse, which may be associated with the decline in Aβ phagocytosis levels.

### 2.2 Knockdown of NgR in microglia decreases amyloid deposition, rescues cognitive deficits, and increases microglial clearance of Aβ in the APP/PS1 transgenic mice

**FIGURE 2:**
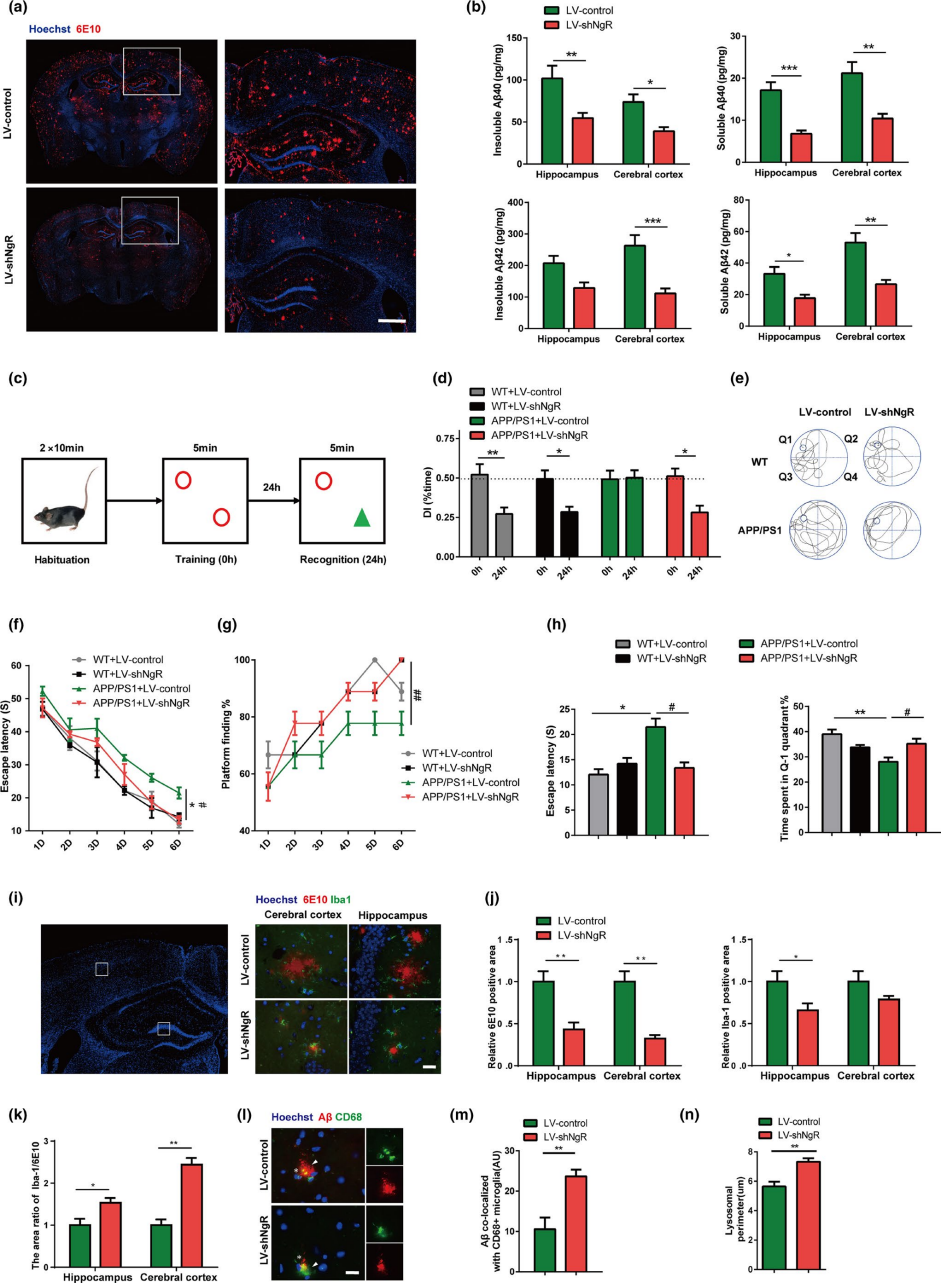
NgR knockdown leads to a strong reduction of amyloid deposition, rescues memory impairment and enhances microglial Aβ phagocytosis in APP/PS1 mice. (a) Brain sections of 14‐month‐old APP/PS1+LV‐control or APP/PS1+LV‐shNgR mice were stained with Hoechst (blue) and 6E10 (red) to detect Aβ deposition. Scale bar: 500 μm. (b) The brain was homogenized and separated Aβ into insoluble and soluble fractions. Amounts of Aβ42 and Aβ40 were measured by ELISA and normalized to the homogenate protein concentration in hippocampal and cortical regions. Statistical analyses were performed using 2‐tailed Student's *t*‐test, *n* = 5–8 mice per group. *p < 0.05; **p < 0.01; ***p < 0.001. (c) Overview of the NOR memory test, with the novel object (green triangle). (d) Recognition memory (recognition session, 24 h) of the old versus new object was assessed as the discrimination index (DI), the ratio of time spent exploring the old object to time spent exploring both objects. Statistical analyses were performed using 2‐tailed Student's *t*‐test, *n* = 9–12 mice per group. *p < 0.05; **p < 0.01. (e‐h) Morris water‐maze analysis of 14‐month‐old mice on 6 consecutive days, including 5 days training test (Days 1–5) and 1 day probe trial test (Day 6). Data were shown as mean ±SEM. Statistical analyses were performed using a two‐way ANOVA with Bonferroni's multiple‐comparison post hoc test, *n* = 9–12 mice per group. *p < 0.05, **p < 0.01 for APP/PS1+LV‐control vs. WT+LV‐control on Day 6; #*p* < 0.05, ##*p* < 0.01 for APP/PS1+LV‐shNgR vs. APP/PS1+LV‐control on Day 6. (e) Representative runs from the probe trials on Day 6. (f) Mean escape latency from Day 1 to Day 6. (g) Percentage of finding the platform from Day 1 to Day 6. (h) Left panel: Mean escape latency during the probe trial on Day 6; Right panel: Percentage of time spent in the target quadrant during the probe trial on Day 6. (i) Coronal sections from 14‐month‐old APP/PS1+LV‐control or APP/PS1+LV‐shNgR mice were stained with Hoechst (blue) for nuclei, 6E10 (red) for Aβ, and Iba‐1 (green) for microglia. Representative images of the cerebral cortex and hippocampus regions were shown. Scale bar, 25 μm. (j) Left panel: Quantification of the Aβ plaque area in brain regions (cerebral cortex and hippocampus); Right panel: Quantification of the Iba‐1+ microglia area in brain regions. (k) The ratio of Iba‐1+ microglia area to 6E10+ Aβ plaque area in brain regions. (l) CD68+ lysosomes (green) containing Aβ immunoreactive content (red) were detected in mice (arrowheads). Scale bars: 20 μm. (m) CD68+ lysosomal content associated with Aβ plaques were quantitatively analyzed. (n) The lysosomal perimeter of microglia. (i‐n) Data were shown as mean ±SEM. Statistical analyses were performed using 2‐tailed Student's *t*‐test. *n* = 5–8 mice per group, and 2 brain sections per animal were used for the analysis. *p < 0.05, **p < 0.01

The APP/PS1 transgenic model exhibits an increase in plaque burden between the ages of 4 and 14 months (Jankowsky & Slunt, 2001). Therefore, in this study, the APP/PS1+LV‐control and APP/PS1+LV‐shNgR animals were examined at the stage of compact and diffuse plaque burden (14 months). A widespread distribution of Aβ plaques stained with 6E10 was found throughout the hippocampus and cerebral cortex of 14‐month‐old APP/PS1+LV‐control mice. By contrast, APP/PS1+LV‐shNgR mice showed a marked reduction in Aβ plaque burden in both regions (Figure 2a). Regarding Aβ peptide concentrations, we found a significantly lower Aβ42 concentration in the sodium dodecyl sulfate (SDS)‐insoluble fractions and RIPA‐soluble fractions of APP/PS1+LV‐shNgR mice compared to APP/PS1+LV‐control mice, in both the hippocampus and cortex. Compared to APP/PS1+LV‐control mice, APP/PS1+LV‐shNgR mice also exhibited significantly lower concentrations of Aβ40 in both RIPA‐soluble fractions and SDS‐insoluble fractions (Figure 2b). In summary, NgR knockdown reduced amyloid plaque deposition and Aβ levels in the APP/PS1 mouse model of AD.

To investigate whether the reduction of amyloid plaque deposition and Aβ levels induced by NgR knockdown is associated with improved memory performance, we assessed spatial memory in 14‐month‐old WT+LV‐control, WT+LV‐shNgR, APP/PS1+LV‐control, and APP/PS1+LV‐shNgR mice using the novel object recognition (NOR) test, which is a memory task that relies on the innate preference of mice for a novel rather than familiar object. Mice were trained as illustrated in Figure 2c. APP/PS1+LV‐shNgR mice performed normally, in contrast to APP/PS1+LV‐control mice, which were not able to distinguish the old from the new object at 24 h (Figure 2d). Next, we used the Morris water‐maze (MWM) test to further confirm these results. Spatial learning and memory in WT+LV‐control and WT+LV‐shNgR mice were unaltered (Figure 2e–h). Compared to WT+LV‐control mice, APP/PS1+LV‐control mice displayed impaired spatial learning, reflected in the time traveled to the platform at Day 6 (Figure 2f, h). NgR deficiency prevented learning and memory dysfunction in APP/PS1 transgenic mice, as shown by the results of the spatial trial on Day 6 (Figure 2g‐h). Thus, APP/PS1+LV‐shNgR mice were protected from the memory dysfunction observed in APP/PS1+LV‐control mice. Our findings support the highly beneficial effect of microglial NgR knockdown with respect to prevention of NOR and MWM memory deficits.

Because Aβ plaques are surrounded by activated microglia in AD (Kamphuis and Orre et al., 2012), we analyzed the colocalization of plaques and microglia in APP/PS1+LV‐control and APP/PS1+LV‐shNgR mice by co‐staining with anti‐Aβ (6E10) and anti‐Iba‐1 antibodies. In APP/PS1 mice, conditional knockdown of NgR in microglia decreased 6E10‐immunoreactive plaques, which was associated with fewer reactive microglia in the hippocampus and cerebral cortex (Figure 2i‐j). Although the relative area of microglia was reduced, the ratio of Iba‐1/6E10 was increased in APP/PS1+LV‐shNgR mice (Figure 2k). CD68, a transmembrane glycoprotein of the lysosomal/endosomal‐associated membrane glycoprotein family, acts as a scavenger receptor for Aβ clearance (de Villiers & Smart, 1999). Notably, more colocalization of Aβ and CD68 was observed in microglial cells in LV‐shNgR mice (Figure 2l‐m), which was associated with larger‐sized CD68+ lysosomes in these cells (Figure 2n). These findings support a primary role of microglial NgR in suppressing the recruitment and activation of microglia, thus leading to the burden of Aβ seen in the APP/PS1 model.

### 2.3 Inhibition of NgR promotes Aβ phagocytosis by upregulating CD36 in primary microglia

**FIGURE 3:**
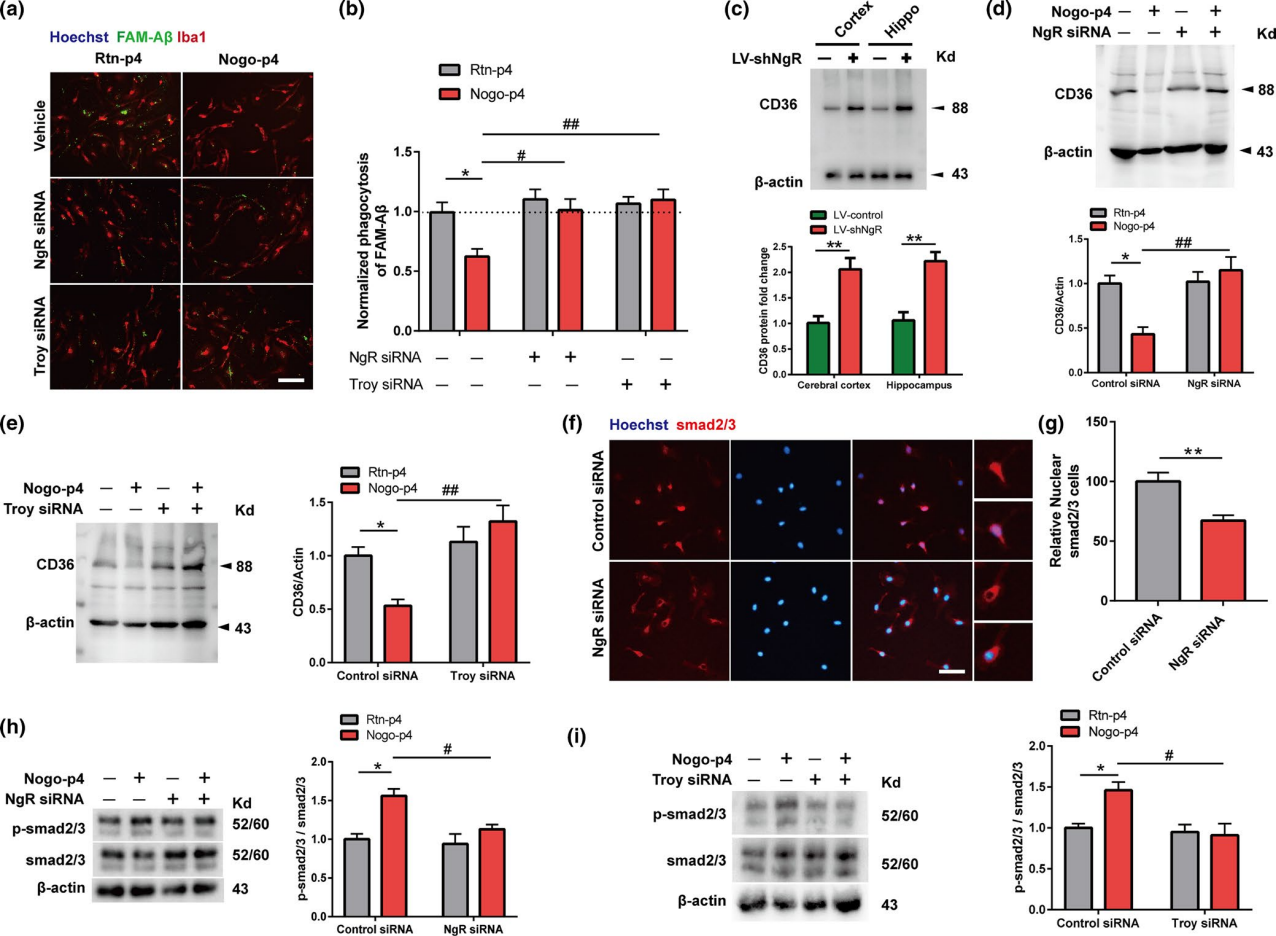
NgR inhibits the phagocytic efficiency of Aβ by suppressing CD36 expression. (a) Before adding Rtn‐p4 (100 μg/ml) or Nogo‐P4 (100 μg/ml) for 12 h, the primary microglia were pretreated with control‐siRNA, NgR‐siRNA/Troy‐siRNA for 24 h to suppress the expression of NgR/Troy. Representative images of primary microglia phagocytosis (Iba‐1, red) after incubated with 0.5 μM FAM‐Aβ (Anaspec, green) for 4 h. Scale bar: 20 μm. (b) The quantification of the phagocytosis assay. *n* = 3wells/group, and *N* = 3 independent experiments (data presented are the average of all experiments). (c) The expression of CD36 protein was detected in cerebral cortex and hippocampus tissues isolated from APP/PS1+LV‐control or APP/PS1+LV‐shNgR mice. *n* = 5–8 mice per group. Statistical analyses were performed using 2‐tailed Student's *t*‐test. (d‐i) The primary microglia were treated with Rtn‐p4 (100 μg/ml) or Nogo‐P4 (100 μg/ml) for 12 h after transfection with control‐siRNA/NgR‐siRNA (d,f‐h) or control‐siRNA/Troy‐siRNA (e,i) for 24 h. (d‐e) The relative CD36 protein expression levels were quantitatively analyzed. (f‐g) Immunostaining of microglia with Smad2/3 (red) and Hoechst (blue). Scale bar, 10 μm. Statistical analyses were performed using 2‐tailed Student's *t*‐test. *n* = 3wells/group, and *N* = 3 independent experiments. (h‐i) The relative p‐Smad2/3 protein expression levels were quantitatively analyzed. (d‐e, and h‐i) Data were shown as the mean ±SEM. *n* = 3–5 samples/group in every experiment, and *N* = 3 independent experiments. Statistical analyses were performed using a two‐way ANOVA with Bonferroni's multiple‐comparison post hoc test, *p < 0.05, **p < 0.01 for control‐siRNA+Nogo‐p4 vs. control‐siRNA+Rtn‐p4; and #*p* < 0.05, ##*p* < 0.01 for NgR‐siRNA/Troy‐siRNA+Nogo‐p4 vs. control‐siRNA+Nogo‐p4

Although NgR physically interacts with APP and Aβ (Park & Gimbel, 2006), and inhibits microglial adhesion and migration to Aβ (Fang & Wang, 2018), little is known regarding the molecular mechanism underlying the direct regulation of Aβ phagocytosis by microglial NgR. To determine how and whether Aβ phagocytosis is directly regulated by microglial NgR, phagocytic efficiency was evaluated by measuring the intracellular levels of FAM‐Aβ in primary microglia purified after treatment with control siRNA or NgR siRNA for 24 h. Troy as a partner of NgR, found to be expressed on the surface of microglial cells (Park & Yiu, 2005), was also silenced by siRNA interference at the same condition. Nogo‐p4 is an agonist of NgR, and Rtn‐p4 (Reticulon 1(Rtn1) non‐inhibitory control/blocking peptide) was used as the negative control. Our data showed that Nogo‐p4 decreased phagocytosed Aβ compared to Rtn‐p4 (Figure 3a‐b). Conversely, both NgR siRNA and Troy siRNA reversed this effect. These data indicate that NgR on microglia can mediate the clearance of Aβ.

CD36, as a pattern recognition receptor on macrophages, mediates the clearance of Aβ, as well as of microbial pathogens (El & Moore, 2003). APP/PS1+LV‐shNgR mice showed greater expression of CD36 compared to APP/PS1+LV‐control mice (Figure 3c). Given that the expression of CD36 was upregulated by conditional knockdown of NgR in microglia in the APP/PS1 mice, we investigated whether NgR could influence Aβ phagocytosis by regulating CD36 expression in primary microglia. NgR siRNA or Troy siRNA treatment significantly improved the protein expression (Figure 3d‐e) of CD36 in microglia after Nogo‐p4 stimulation, indicating that knockdown of NgR inhibited the suppressive effect of Nogo‐p4 on the expression of CD36. However, the protein levels of RAGE and Trem2, as the microglial surface receptors genetically linked to Aβ phagocytosis, were not affected by NgR siRNA (Figure S1a‐b). Aβ internalization in microglia is increased by blockade of TGF‐β‐Smad2/3 signaling (Liu & Liu, 2014; Town & Laouar, 2008), and TGF‐β downregulated the scavenger receptor CD36 through Smad2/3 transcription (Rustenhoven & Aalderink, 2016). However, whether NgR modulates CD36 by mediating Smad2/3 signaling has not been investigated. Immunofluorescence staining showed that NgR siRNA decreased Smad2/3 nuclear translocation to microglia (Figure 3f‐g). Furthermore, NgR siRNA or Troy siRNA significantly attenuated the phosphorylation of Smad2/3 in microglia that was increased by Nogo‐p4 (Figure 3h‐i). The results indicated that Nogo‐P4 bound to the NgR and triggered the activation of the Smad2/3 signaling. Collectively, these data showed that Smad2/3 and CD36 are essential for Aβ phagocytosis in primary microglia regulating by NgR.

### 2.4 Activation of ROCK‐Smad2/3 signaling is necessary for the decreased CD36 expression induced by NgR in microglia

**FIGURE 4:**
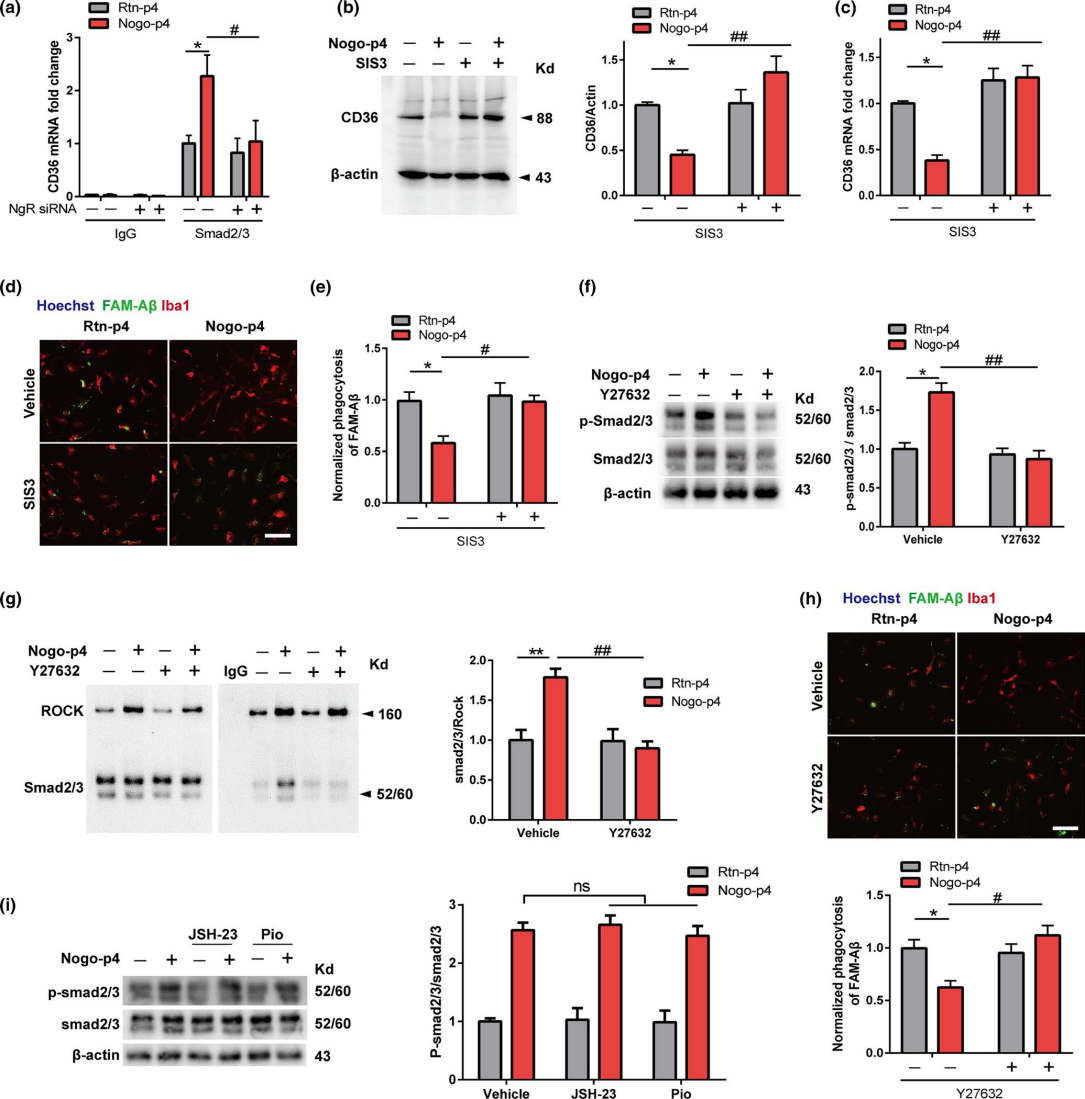
NgR inhibits CD36 expression by activating ROCK‐Smad2/3 signaling in microglia. (a) The primary microglia were treated with Rtn‐p4 (100 μg/ml) or Nogo‐P4 (100 μg/ml) for 12 h after transfection with control‐siRNA or NgR‐siRNA for 24 h, and then, the Chip‐qPCR analysis was used to measure Smad2/3 occupancy at the Smad2/3 binding site on CD36 promoter. (b‐e) The primary microglia were treated with Rtn‐p4 (100 μg/ml) or Nogo‐P4 (100 μg/ml) for 12 h after treatment with PBS or SIS3 (10 μM) for 12 h. (b‐c) The relative CD36 protein and mRNA levels were quantitatively analyzed. (d) Representative images of phagocytosed by primary microglia phagocytosis (Iba‐1, red) after incubated with 0.5μM FAM‐Aβ (Anaspec, green) for 4 h. Scale bar: 20 μm. (e) The quantification of the phagocytosis assay. *n* = 3wells/group, and *N* = 3 independent experiments. (f‐h) The primary microglia were treated with Rtn‐p4 (100 μg/ml) or Nogo‐P4 (100 μg/ml) for 12h after treatment with PBS or Y27632 (50 μM) for 30 min. (f) The relative p‐Smad2/3 protein expression levels were quantitatively analyzed. (g) Co‐IP assay confirms the protein binding between ROCK and Smad2/3. Total protein was extracted and subjected to INPUT and IP study with indicated antibodies. (h) Representative images of primary microglia phagocytosis (Iba‐1, red) after incubated with 0.5μM FAM‐Aβ (Anaspec, green) for 4 h and the quantification of the phagocytosis assay. Scale bar: 20 μm. *n* = 3wells/group, and *N* = 3 independent experiments. (i) The primary microglia were treated with Rtn‐p4 (100 μg/ml) or Nogo‐P4 (100 μg/ml) for 12 h after treatment with PBS, JSH‐23 (10 μM) or Pioglitazone (1 μM) for 6 h, and the relative p‐Smad2/3 protein expression levels were quantitatively analyzed. Values were reported as mean±SEM. *n* = 3–5 samples/group in every experiment, and *N* = 3 independent experiments. Statistical analyses were performed using a two‐way ANOVA with Bonferroni's multiple‐comparison post hoc test, *p < 0.05, **p < 0.01 for vehicle+Nogo‐p4 vs. vehicle+Rtn‐p4; and #*p* < 0.05, ##*p* < 0.01 for NgR‐siRNA/SIS3/Y27632+Nogo‐p4 vs. control‐siRNA/vehicle+Nogo‐p4; ns, not significant

We then checked whether NgR preserved CD36 in a Smad2/3‐dependent manner. The ChIP assay showed that Nogo‐p4 increased the binding of Smad2/3 and the CD36 promoter, which was inhibited by NgR siRNA (Figure 4a). To test whether microglial Aβ internalization can be increased by blocking the Smad2/3 signaling, we used the Smad2/3‐specific inhibitor SIS3. Interestingly, the CD36 protein and mRNA expression levels in the primary microglia were increased after SIS3 treatment (Figure 4b‐c). The pharmacological blockage of Smad2/3 by SIS3 clearly reversed the Nogo‐p4‐mediated inhibited effect on Aβ phagocytosis (Figure 4d‐e), suggesting that NgR affects microglial phagocytosis of Aβ and CD36 expression by a mechanism dependent on the activation of Smad2/3.

Nogo‐p4 binding to NgR inhibits the adhesion and migration of microglia to Aβ through the ROCK pathway (Fang & Wang, 2018); thus, we determined whether NgR enhanced Smad2/3 signaling in a ROCK‐dependent manner. Y27632, an inhibitor of ROCK, increased the phosphorylation of Smad2/3, which was reduced by Nogo‐p4 stimulation in the primary microglia (Figure 4f). After microglia had been pre‐incubated with Nogo‐p4, the interaction between ROCK and Smad2/3 was significantly disrupted, which was reversed by Y27632 treatment (Figure 4g). Furthermore, Nogo‐p4 decreased phagocytosed Aβ compared to Rtn‐p4, which was reversed this effect after Y27632 treatment (Figure 4h). These results suggest that NgR increases Smad2/3 signaling through the ROCK pathway. The nuclear factor‐kappa B (NF‐кB) pathway also plays a role in regulating Smad2/3 signaling and influences Aβ phagocytosis (Liu & Liu, 2014), while the peroxisome proliferator‐activated receptor‐r (PPARг) pathway increases Aβ phagocytosis, an effect mediated by the upregulation of CD36 expression (Yamanaka & Ishikawa, 2012). To determine whether the NF‐кB or PPARг pathway regulates Smad2/3 signaling induced by Nogo‐p4, we used the NF‐кB activation inhibitor JSH‐23 and the PPARг agonist pioglitazone, respectively (Figure 4i). Neither JSH‐23 nor pioglitazone affected the phosphorylation of Smad2/3 after Nogo‐p4 stimulation. Of note, these data suggest that NgR decreases Aβ phagocytosis through ROCK‐Smad2/3‐CD36 signaling, but not the NF‐кB or PPARг pathway.

### 2.5 NgR decreases microglial Aβ uptake in the AD model after intra‐hippocampal fAβ injection

**FIGURE 5:**
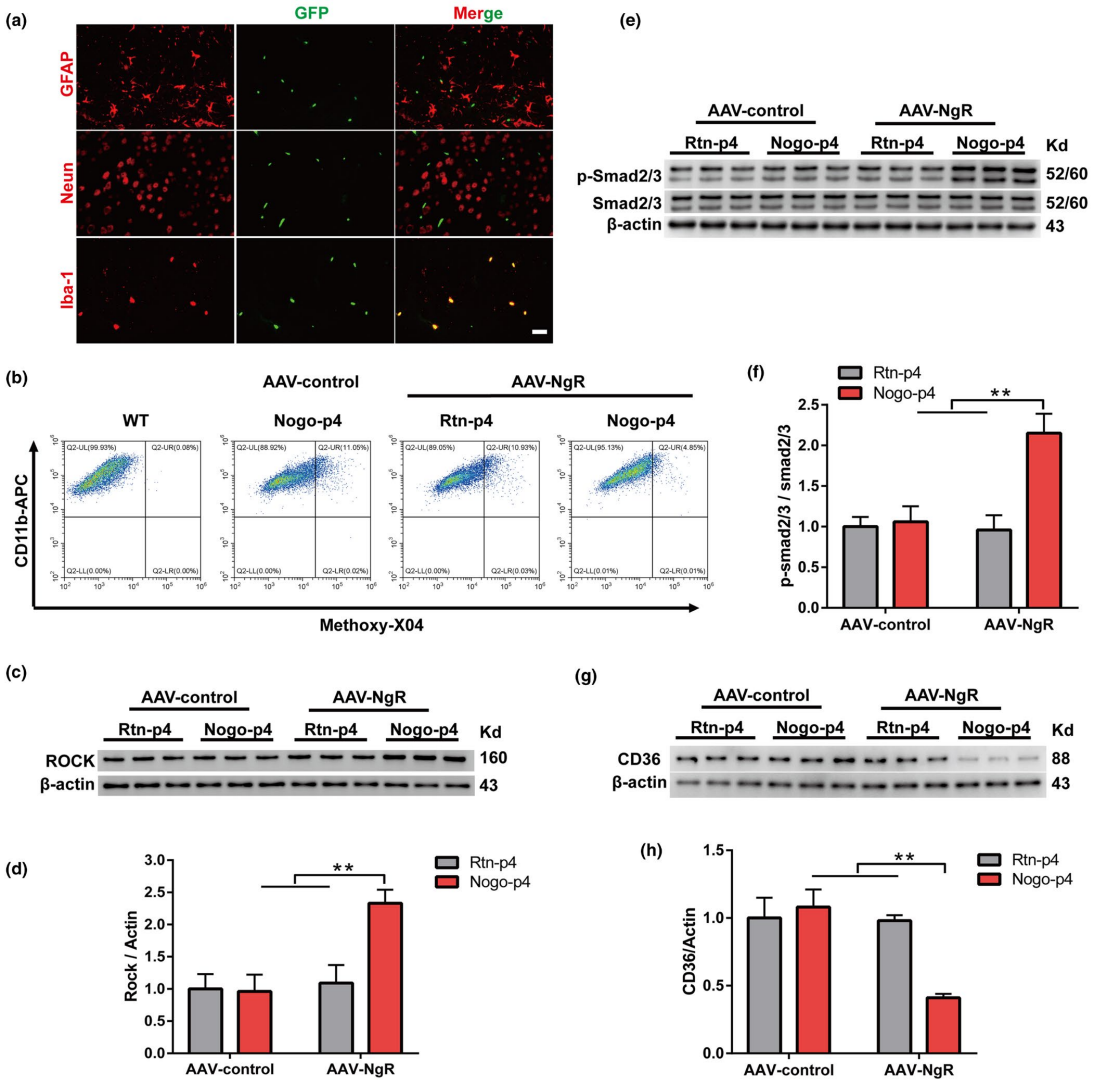
AAV‐NgR treatment causes a decrease in microglial Aβ phagocytosis after intra‐hippocampal fAβ injection (a) Confocal microscope images from brain sections of mice treatment with tamoxifen‐induced GFP (green) stained with astrocytes markers GFAP, neuronal markers NeuN, and microglia markers Iba‐1(red) 30 days after injection. Scale bar, 50 μm. (b) Representative FACS dot plots showing the microglial phagocytosis of Aβ in intra‐hippocampal fAβ injection mice. (c‐h) ROCK (c), P‐Smad2/3 (e), and CD36 (g) proteins from the hippocampus were analyzed by Western blotting 2 days after Rtn‐p4 or Nogo‐p4 injection to the Cx3cr1CreER mice after treatment with AAV‐ control or AAV‐NgR for another 26 days. The quantitation of Western blots in ROCK (d), P‐Smad2/3 (f), and CD36 (h). Data were shown as mean ±SEM. *n* = 6–9 mice per group. Statistical analyses were performed using a two‐way ANOVA with Bonferroni's multiple‐comparison post hoc test, **p < 0.01 for AAV‐NgR+Nogo‐p4 vs. all three other groups

To better understand the role of NgR in microglial Aβ uptake *in vivo*, WT and Cx3cr1CreER mouse were administered adeno‐associated virus (AAV)‐control or AAV‐DIO‐NgR (AAV‐NgR), with or without Nogo‐p4, and subsequently received fibrillar Aβ42 (fAβ) injections into the hippocampus at the age of 2 months. To investigate the efficiency of tamoxifen, we first analyzed the pattern of hippocampal green fluorescent protein (GFP) fluorescence in mice 30 days after tamoxifen treatment. In Cx3cr1CreER mice administered tamoxifen, GFP+staining expressed only of microglial cells (Iba‐1+) in brain sections, but not astrocytes (GFAP+) or neurons (NeuN+) (Figure 5a). Subsequently, to test whether it is microglia‐specific expression of NgR, NgR was stained in the brain sections after AAV‐NgR administration. As shown in Figure S2a, almost all of the GFP+cells colocalized with NgR+cells, which revealed that microglia cells were the primary cell type transfected by the AAV‐NgR vectors.

We intraperitoneally administered methoxy‐X04 at 3 h prior to microglial isolation and analyzed the proportion of methoxy‐X04‐positive microglial cells by flow cytometry to further test the effects of NgR on Aβ uptake. A significant decrease in the proportion of methoxy‐X04‐positive microglial cells was observed in the AAV‐NgR+Nogo‐p4 mouse group versus the AAV‐NgR+Rtn‐p4 or AAV‐control+Nogo‐p4 groups, respectively (Figure 5b), further demonstrating that NgR decreased the microglial phagocytosis of Aβ *in vivo*. We further examined the effects of ROCK‐Smad2/3‐CD36 signaling on Aβ uptake in this model. In contrast to the increased expression of ROCK (Figure 5c‐d) and improved phosphorylation of Smad2/3 (Figure 5e‐f), the AAV‐NgR+Nogo‐p4 group showed decreased expression of CD36 compared to the AAV‐NgR+Rtn‐p4 or AAV‐control+Nogo‐p4 groups (Figure 5g‐h). Therefore, these results demonstrate that NgR acutely decreased microglial Aβ uptake in the AD model after intra‐hippocampal fAβ injection by regulating ROCK‐Smad2/3‐CD36 signaling.

## 3 DISCUSSION

AD is a neurodegenerative brain disorder characterized by the formation of Aβ plaques, predominantly in hippocampal and cortical regions (Mattson, 2004). Impaired cerebral Aβ clearance has been proposed as the cause of sporadic AD in vast majority of cases (Mawuenyega & Sigurdson, 2010). Microglial activities are critical in the defense against the development of neurodegenerative disease. In AD, activated microglia are in close proximity to Aβ deposits, and produce a wide range of cytokines and chemokines (Rogers & Lue, 2001). The first responders to Aβ accumulation are microglia, which are recruited to amyloid plaques. Furthermore, clearance of Aβ is controlled by complex mechanisms, and dysregulation thereof is suggested to be a major reason for disease onset (Wildsmith & Basak, 2012). Aging is one of the major risk factors for the development of various diseases, including AD, and recent studies have noted a steady decline in normal microglial functions in aging and AD (Johansson & Woodling, 2015). Interestingly, the Aβ‐clearing capacity of microglia in APP/PS1 mice is impaired with aging, as indicated by the reduced expression of Aβ‐binding receptors and Aβ‐degrading enzymes (Hickman & Allison, 2008), but the exact mechanism is unclear. In this study, we found that the effects of microglial Aβ phagocytosis were reduced, in contrast to the upregulation of NgR seen in aging microglia from APP/PS1 mice. This interesting age‐dependent effect suggests that microglial NgR maybe exerted harmful effects in response to Aβ phagocytosis and accumulation in APP/PS1 mice.

We all know that NgR plays an important role not only in inhibition of neurite/axon growth (Fournier & GrandPre, 2001), but also in AD pathogenesis (Masliah et al., 2010; Park & Gimbel, 2006). It has been demonstrated that Nogo‐A expression is increased in the hippocampus of patients with AD and is also localized in senile plaques around amyloid deposits (Gil & Nicolas, 2006). In addition, deleting Nogo improves the learning and memory deficits of APP transgenic mice (Masliah et al., 2010). Furthermore, the expression of NgR and downstream signaling proteins are elevated in patients with AD and aged rats with deficits of spatial cognition (VanGuilder & Sonntag, 2013; Zhu & Guo, 2007), and neuronal overexpression of NgR impairs cognitive function in APP/PS1 mice (Karlsson et al., 2013). However, Park and Gimbel found a protective role for NgR in AD, in which NgR, presumably on the neuronal cell surface, interacts with APP on the cell membrane to block its processing by secretases, thereby reducing Aβ, amyloid plaque deposition, and dystrophic neurites (Park & Gimbel, 2006). Thus, above studies imply that the role of NgR in AD pathology is very complicated. In our previous and other researcher's studies, NgR is not only expressed on the neuron (Fournier & GrandPre, 2001), but also expressed on the microglia and astrocyte (Wang & Sun, 2018; Yan & Zhou, 2012). In view of the distribution of the NgR+cells around the periphery of the plaques (VanGuilder & Sonntag, 2013; Zhu & Guo, 2007) and the colocalization of NgR and microglia was detected in hippocampus and cerebral cortex in the 8‐month‐old AD transgenic mice (Fang & Yao, 2016), we dissected the role of NgR expressed on the microglia in AD transgenic model mice. We found that the adhesion and migration of microglia to Aβ were inhibited by Nogo/NgR pathway *in vitro* and *in vivo* (Fang & Wang, 2018) and blocking Nogo/NgR signal pathway in microglia improved the neuroinflammatory environment in APP/PS1 mice and alleviated the formation of Aβ plaques and tau phosphorylation in APP/PS1 transgenic mice (Fang & Yao, 2016). The work by Jiang et al (Jiang et al., 2020) showed that NgR plays a critical role in APP processing through Rho/ROCK signaling pathway, and NgR knockdown in the perforant path reduces the APP level and Aβ production, which might account for the improved synaptic and cognitive function in the APP/PS1 mice. Differing from the research on Aβ production by Jiang (Jiang et al., 2020) in perforant path, the functions of NgR in microglial Aβ phagocytosis were explored in this paper. Our results showed that NgR participated in Aβ pathology by modulating the microglial phagocytosis of Aβ in APP/PS1 mice. Conditional knockdown of NgR in microglia reduced the amyloid burden and improved learning and memory by enhancing Aβ phagocytosis in the brain. In addition, we elucidated the molecular mechanism by which NgR deficiency promotes the phagocytosis of Aβ by upregulating ROCK‐Smad2/3‐CD36 signaling in microglia, both *in vitro* and *in vivo*. In conclusion, NgR expressed in microglia impaired the clearance of Aβ and accelerated Alzheimer's‐like disease progression. Therefore, these results suggest that NgR has different roles mainly based on its expression pattern in microglia and neuronal cells and imply the function of NgR in the pathogenesis of AD is relatively complicated and remains to be elucidated furthermore.

To better understand the role of microglial NgR in response to Aβ phagocytosis *in vivo*, we used the LV‐shNgR technique under control of the CD11b promoter to conditionally delete the protein of interest in APP/PS1 transgenic mice (Gowing & Vallieres, 2006), in which the NgR gene was decreased only in endogenous microglial cells, thereby excluding any confounding effects of neuronal NgR (which reportedly regulate Aβ production) (Zhou & Hu, 2011). We observed strong attenuation of Aβ plaque formation and strikingly diminished Aβ peptide concentrations in brain tissue from NgR‐deficient APP/PS1 mice, which correlated with less severe behavioral impairment, illustrating the critical role of NgR in the improvement of AD‐like pathology in this model. Furthermore, more CD68+ phagocytic microglial cells in APP/PS1 mice contain amyloid plaques after LV‐shNgR treatment, suggesting that a deficiency in NgR restores the lysosomal activity of microglia and promotes Aβ clearance.

We were interested in determining the mechanisms underlying the ability of NgR to attenuate Aβ phagocytosis. CD36 is an important receptor for microglial Aβ phagocytosis (Coraci & Husemann, 2002). Therefore, we tested whether knockdown of NgR using siRNA would result in a functional improvement in CD36 expression and, subsequently, Aβ phagocytosis. The results showed that NgR knockdown induced the upregulation of CD36, and subsequently enhanced phagocytosis of Aβ. The NF‐кB pathway plays a role in regulating CD36 expression and influences Aβ phagocytosis (Liu and Liu et al., 2014), while PPARг pathway activation increases Aβ phagocytosis (an effect mediated by upregulated CD36 expression) (Yamanaka & Ishikawa, 2012). However, in our study, neither the NF‐кB nor PPARг pathway played a role in NgR knockdown‐induced upregulation of CD36. Moreover, we used the ChIP assay to determine the mechanism by which NgR regulates the expression of CD36, and found that NgR siRNA treatment increased the binding of Smad2/3 to the CD36 promoter; in other words, NgR inhibited the transcription of CD36 in microglia regulated by Smad2/3. Similarly, it has been reported that Smad2/3 signaling downregulates the scavenger receptor CD36 and reduces Aβ phagocytosis (Town and Laouar et al., 2008; Rustenhoven and Aalderink et al., 2016). Our previous study showed that Nogo‐66 binding to NgR can inhibit the adhesion and migration of microglia through the ROCK pathway in AD (Fang and Yao et al., 2016). We hypothesized that ROCK might be a key factor in the intracellular signal transduction of NgR, so we tested the interaction between ROCK and Smad2/3 using Co‐IP assays. Nogo‐p4 significantly disrupted the interaction between ROCK and Smad2/3, which was reversed by Y27632 treatment. Ultimately, we demonstrated that NgR deficiency enhanced microglial Aβ phagocytosis through ROCK‐Smad2/3‐CD36 signaling. To better understand the role of NgR *in vivo* microglial Aβ uptake, we used intra‐hippocampal fAβ injection mouse models that recapitulate the acute aspects of microglial responses to Aβ peptides (Johansson & Woodling, 2015; Krauthausen & Kummer, 2015). We found that NgR decreased microglial Aβ uptake in the AD model after intra‐hippocampal fAβ injection via regulating ROCK‐Smad2/3‐CD36 signaling. Taken together, these data suggest that NgR decreases Aβ phagocytosis through ROCK‐Smad2/3‐CD36 signaling.

In summary, we showed that NgR participates in Aβ pathology by modulating the microglial phagocytosis of Aβ in APP/PS1 mice. Conditional knockdown of NgR in microglia reduces the amyloid burden and improves learning and memory, by enhancing Aβ phagocytosis in the brain. In addition, we elucidated the molecular mechanism by which NgR deficiency promotes the phagocytosis of Aβ by upregulating ROCK‐Smad2/3‐CD36 signaling in microglia, both *in vitro* and *in vivo*. Thus, NgR may play an indispensable role in the reduction of Aβ deposits and has potential as a therapeutic target for AD.

## 4 EXPERIMENTAL PROCEDURES

### 4.1 Animals

C57BL/6J mice were obtained from Vitalriver (Beijing, China). Cx3Cr1^CreER^ mice were obtained from Soochow University. APP/PS1 transgenic mice were purchased from the animal model center of Nanjing University (Nanjing, China), and the mice were generated from the B6C3‐Tg (APPswe, PSEN1dE9) 85Dbo/J double transgenic mouse line (Stock # 004462) provided by the National Jackson Animal Center (Bar Harbor, Maine, USA). Only male mice were used in the experiments to minimize the effect of hormones in the behavioral analysis (Lee et al., 2021). The mice were housed in a controlled temperature environment under a 12‐h light/dark cycle and had a libitum access to standard food and water.

All animal experiments were carried out in accordance with the US National Institute of Health (NIH) Guide for the Care and Use of Laboratory Animals published by the US National Academy of Sciences, and the Provision and General Recommendation of Chinese Experimental Animals Administration Legislation and approved by the Science and Technology Department of Jiangsu Province (SYXK (SU) 2016–0011). All experimental procedures were approved by Institutional Animal Care and Use Committee (IACUC) of the Nanjing Medical University Experimental Animal Department.

### 4.2 Microglia from adult mice

3‐month‐old and 8‐month‐old C57BL/6J mice and APP/PS1 mice were anesthetized with chloral hydrate (100 mg/kg, i.p.), and perfused with ice‐cold D‐Hanks’ balanced salt solution to wash away all contaminating blood cells from the brain. The cerebellum and meninges were removed from the brains. Then, the tissue was dissected and dissociated in an enzymatic solution (116 mM NaCl, 5.4 mM KCl, 26 mM NaHCO_3_, 1 mM NaH2PO_4_, 1.5 mM CaCl_2_, 1 mM MgSO_4_, 0.5 mM EDTA, 25 mM glucose, 1 mM cysteine, and 20 units/ml papain, Sigma) at 37°C and 5% CO_2_ and continuously stirred for 90 min. Next, the enzymatic reaction was quenched by the addition of 20 ml of 20% FBS in D‐Hank's. After centrifugation at 200 g for 7 min at room temperature, the cells were dissociated in 0.05 mg/ml DNase I (Sigma) in D‐Hank's and incubated for 5 min at room temperature. Then, the tissues were gently disrupted and filtered through a 70 μm‐cell strainer (Corning, New York, USA). To remove the myelin, cells were suspended in 20 ml 20% stock isotonic Percoll (SIP =v/v ratio: 9/10 Percoll (GE Healthcare, Princeton, NJ, USA) +1/10 D‐Hank's 10×) in D‐Hank's and centrifuged for 20 min at 500 g with slow acceleration and no brake. The supernatant containing the myelin was removed, and the pelleted cells were washed with D‐Hank's. Then, the cells were incubated with anti‐mouse CD11b‐coated microbeads (Miltenyi Biotec) for 15 min at 4°C. The cells were washed with washing buffer (0.5% BSA, 2 mM EDTA in PBS) to remove unbound beads. The cells were resuspended in washing buffer and passed over a magnetic MACS Cell Separation column (Miltenyi Biotec), and the column was rinsed twice with washing buffer. CD11b+ microglia were eluted by removing the column from the magnetic holder and the pushing washing buffer through the column with a plunger. The cells were centrifuged and washed with DMEM/F12 containing 10% FBS. Approximately 95% of these cells were positive for CD11b, a marker for microglia cell types.

### 4.3 Preparation of fibrillar Aβ

FAM‐labeled Aβ1‐42 (FAM‐Aβ, AnaSpec Inc., San Jose, California, USA) lyophilized powder was dissolved to 1 mg/200 μl in 100% hexafluoroisopropanol and aliquoted into 10 μl portions, dried in a vacuum centrifuge, and stored at −20°C. To fibrillize, FAM‐Aβ peptides were resuspended in sterile ddH_2_O followed by incubation for 1 week at 37°C.

### 4.4 Microglial *in vitro* phagocytosis of FAM‐labeled fAβ

Microglial FAM‐Aβ phagocytosis reader assay was applied as described previously (Floden & Combs, 2006). Briefly, 5 × 10^4^ cells/well were seeded and incubated with 0.5 μM fibrillar FAM‐Aβ for 4 hours. Afterward, the medium was removed, and extracellular FAM‐Aβ fluorescence signal was quenched with 0.2% trypan blue in PBS (pH 4.4) for 1 min. Fluorescence intensity was measured at 485 nm excitation/535 nm emission using a fluorescence plate reader (Infinite 200 M, Tecan). To compensate for different cell counts, results were normalized to the Hoechst Dye 33342 (Sigma Aldrich) nuclear stain signals.

### 4.5 Western blot analysis

Equal amounts of protein (40 µg) were separated electrophoretically using denaturing gels and transferred to nitrocellulose membranes. Subsequently, the membranes were blocked for 1 h at room temperature with 5% BSA in TBST, and incubated with following primary antibody overnight at 4°C: mouse anti‐β‐actin monoclonal antibody (1:2000; Santa Cruz Biotechnology, #sc‐47778), rabbit anti‐CD36 polyclonal antibody (1:1000; Novus Biologicals, #NB400‐144), rabbit anti‐NgR antibody (1:1000; Merck Millipore, #AB 15138), rabbit anti‐ROCK antibody (1:1000; Cell Signaling Technology Inc., #4035), rabbit anti‐Smad2/3 antibody (1:1000; Cell Signaling Technology Inc., #8685), and rabbit anti‐phospho‐Smad2/3 antibody (1:1000; Cell Signaling Technology Inc., #8828). After washed with TBST thrice, the membranes were incubated with horseradish peroxidase‐conjugated secondary antibodies anti‐mouse IgG (1:10000; Sigma) or anti‐rabbit IgG (1:5000; Cell Signaling Technology Inc.). The immunoreactive bands were visualized using chemiluminescence reagents (ECL; Millipore) and captured with Bio‐Rad Gel Doc XR documentation system. Pixel density of bands was performed using Quantity One software.

### 4.6 Quantitative RT‐PCR

Total RNAs were extracted from primary microglia using TRIzol reagent (Life Technologies, USA) according to the manufacturer's instructions. RNA concentrations were equalized and converted to cDNA using Hiscript Ⅱ reverse transcriptase kit (Vazyme, Nanjing, China). Gene expression was measured by quantitative PCR system (Roche, Basel, Switzerland) using SYBR‐green (Roche, Basel, Switzerland). The amount of double‐stranded PCR product synthesized in each cycle was measured by detecting the SYBR green dye, which binds to double‐stranded DNA. Threshold cycle (Ct) values for each test gene from the replicate PCRs were normalized to the Ct values for GAPDH RNA control from the same cDNA preparations. Transcription ratios were calculated as 2 ^(ΔCt)^, where ΔCt is the difference between Ct (GAPDH) and Ct (test gene). In addition, we calculated the fold change expression of target genes to the indicated control group. Triplicate PCRs were performed for each of three independently purified RNA samples.

The following PCR primer sequences were used for detecting transcriptions: GAPDH F: 5′‐GGTGAAGGTCGGTGTGAACG‐3′, R: 5′‐CTCGCTCCTGGAAGATGGTG‐3′; NgR F: 5′‐ AGCCCAA GGTAACAACAAGC‐3′, R: 5′‐ GCAGCCACA GGATAGTGAGAT‐3′; CD36 F: 5′‐GCCAGT CGGAGACATGCT TA‐3′, R: 5′‐AT TGAGTCCTGGGG CTCCTG‐3′; the primers were synthesized by Nanjing Genscript (Nanjing, China).

### 4.7 Intracerebral Administration of lentiviral Vectors

At the age of 12 months, when the deposits are readily detected in the brain, APP/PS1 mice and their WT littermates were anesthetized with isoflurane and placed in a stereotactic frame. APP/PS1 mice were administered a lentivirus vector encoding shNgR under control of the CD11b promoter (LV‐shNgR), bilaterally in the hippocampus and cortex, and subsequently sacrificed at 14 months of age. Before injections, lentiviral vectors were diluted with sterile PBS (pH 7.4) to achieve a titer of 1.71 × 10^9^. Viral preparations in 2‐μL volume were injected bilaterally into the hippocampus (±3.2 mm medial/lateral, −2.7 mm anterior/posterior, and −2.7 mm dorsal/ventral from the bregma) and the cerebral cortex (±2.5 mm medial/lateral, −2.0 mm anterior/posterior, and −1.0 mm dorsal/ventral from the bregma). The preparation was injected with a speed of 0.5 μl/min over a period of 4 min by using a Hamilton 5‐μL syringe and a 27 G needle. Before waking, mice were allowed to recover in a heated chamber.

In the brain of 14‐month‐old APP/PS1+LV‐shNgR mice, Western blotting showed that the amount of NgR in CD11b+ cells isolated from APP/PS1+LV‐shNgR mice was significantly lower than that in cells isolated from APP/PS1+LV‐control mice in the hippocampus and cortex (Figure S3a‐b). Similarly, qPCR analysis showed that the level of NgR transcripts in CD11b+ brain cells from APP/PS1+LV‐shNgR mice was 39.30 ± 5.59% of the level of NgR transcripts from APP/PS1+LV‐control mice (Figure S3c). By contrast, in CD11b‐ brain cells, including neurons, astrocytes, and oligodendrocytes, the levels of general NgR transcripts were not affected by LV‐shNgR (Figure S3c).

### 4.8 Aβ ELISA

Levels of Aβ peptides were quantified using human Aβ1‐40 and Aβ1‐42 ELISA kits (Millipore) according to the manufacturer's protocol. Snap‐frozen forebrain hemispheres were homogenized in PBS with protease inhibitor mixture (Sigma). Protein was extracted in RIPA (25 mM Tris‐HCl, pH 7.5, 150 mM NaCl, 0.5% sodium desoxycholate, 1% NP‐40, and 0.1% SDS) for 30 min on ice. After centrifugation at 100,000 g for 30 min at 4°C, the resulting supernatant (RIPA‐soluble fraction) was saved, and the pellet was sacrificed in 25 mM Tris‐HCl, pH 7.5, and 2% SDS (RIPA‐insoluble fraction). RIPA and SDS fractions were analyzed for Aβ1‐40 and Aβ1‐42 peptide concentrations. Samples were analyzed in duplicate. Results were normalized on the basis of the sample's protein concentration.

### 4.9 Morris water‐maze test (MWM)

MWM test was performed to detect spatial memory as previously described (Krauthausen & Kummer, 2015). Spatial memory testing was conducted in a pool consisting of a circular tank (1 m diameter) filed with opacified water at 24°C. The water basin was dimly lit (20–30 lux) and surrounded by a white curtain. The maze was virtually divided into 4 quadrants, 1 containing a hidden platform (15 × 15 cm) present 1.5 cm below the water surface. Mice were trained to find the platform using 3 extra maze cues placed asymmetrically as spatial references. Mice were placed into the water in a quasi‐random fashion to prevent strategy learning. Mice were allowed to search for the platform for 60 s and were placed onto it manually if they did not reach the platform in the allotted time. Mice were allowed to stay on the platform for 15 s before the initiation of the next trial. After 4 trials, mice were dried and placed back into their home cages. Mice were trained for 5 consecutive days with 4 trials per day. In spatial probe trials (Day 6), which were conducted 24 h after the last training session, the platform was removed and mice were allowed to swim for 60 s. Data are given as percentage of time spent in quadrant Q1, where the platform was located previously, which was compared with the average time the animals spent in the remaining quadrants. All movements were recorded by a computerized tracking system that calculated distances moved and latencies required for reaching the platform.

#### 4.9.1 Novel object recognition test (NOR)

The NOR task, based on the ability of mice to show preference for novel versus familiar objects when allowed to explore freely. NOR was performed during the light cycle. Mice were individually habituated to an open arena (50 cm ×50 cm, dim light, 24°C) on Day 1. During the subsequent training session, 2 identical objects were placed into the arena, and exploratory behavior was monitored for 5 min. On Day 2, mice were placed back into the same arena, in which one of the objects used during training was replaced by a novel object of similar dimensions but a different shape/color, and exploratory behavior was monitored for 5 min. Exploration behavior was assessed by calculating DI, the ratio of time spent exploring the old object to time spent exploring both objects (expressed as a percentage). A DI of ~50% is associated with correct training and no object preference; a significant decrease in DI is characteristic of recognition of the novel object. To evaluate memory, comparisons were made for each group between the recognition (24 h) and training (0 h) sessions.

#### 4.9.2 Immunohistochemistry

Coronal cryosections (20 μm) of perfused mouse brains were used for immunohistochemistry. Sections obtained were stored in 0.1% NaN_3_ and PBS in a cold room. For immunohistochemistry, sections were treated with 50% methanol for 15 min. Then, sections were washed three times for 5 min in PBS and blocked in 3% BSA, 0.1% Triton X‐100, and PBS (blocking buffer) for 30 min, followed by overnight incubation with the primary antibody in blocking buffer. Next, sections were washed three times in 0.1% Triton X‐100 and PBS and incubated with Alexa Fluor 488‐conjugated or Alexa Fluor 594‐conjugated secondary antibodies (1:500; Invitrogen) for 90 min, washed three times with 0.1% Triton X‐100 and PBS for 5 min. Finally, the sections were mounted on glasses in tap water and embedded. The following primary antibodies were used with respective concentrations: rabbit anti‐ionized calcium‐binding adapter molecule 1 (Iba‐1; 1:500; 019–19741, Wako), and rat anti‐CD68 (1: 400; MCA1957, Bio‐Rad), mouse anti 6E10 (1:500; SIG‐39300, Covance). Fluorescence microscopy was done on an BX61 equipped with a disk‐spinning unit (Olympus) or an A1‐MP (Nikon) laser‐scanning microscope, and images were processed in Cell‐P (Olympus) or NIS elements (Nikon).

#### 4.9.3 Primary mouse neuronal culture

Primary microglia were prepared from cerebral cortex of neonatal C57BL/6J mice as previously described. Briefly, neonatal mice were ice‐anaesthetized and decapitated. After removing the meninges from the brains, the cortex was enzymatically dissociated (0.25% trypsin‐EDTA, Sigma). Then, cells were suspended in Dulbecco's modified Eagle's medium/nutrient mixture F‐12 (DMEM/F12, Gibco) supplement with 10% FBS (Gibco) and seeded into poly‐L‐lysine (PLL, 0.01 mg/ml, Sigma) pre‐coated T75 tissue culture flasks. Flasks were incubated at 37 °C and 5% CO_2_ until mixed glial cultures were completely confluent. After 2 weeks in culture, cells were isolated by gently shaking of the flask. Microglia in the medium were collected and seeded into new dishes or plates with culture media.

#### 4.9.4 Small interfering RNA knockdown

All electroporation experiments were performed using a Neon system and a Neon transfection kit according to the manufacturer's instructions (Invitrogen, California, USA).

#### 4.9.5 Chromatin Immunoprecipitation (ChIP) Assays

ChIP assay was performed using a ChIP assay kit (Upstate Biotechnology). Mouse primary microglia fixed with 1% formaldehyde/phosphate‐buffered saline, and sonicated to obtain 500‐bp to 1,000‐bp DNA fragments. Chromatin was immunoprecipitated with 5 mg of anti‐Smad2/3 (1:100; #8685, Cell Signaling Technology Inc.) or rabbit IgG. The immunoprecipitated DNA was amplified with a promoter pair specific for the CD36 promoter (F,5’‐ GGGGAAACTCAGCAAGTCAG −3’ and R, 5’‐ AGTGTCAGAT CCCAGTGG −3’).

#### 4.9.6 AAV administration and establishment of the AD model of intra‐hippocampal fAβ

As showed in Figure S4, tamoxifen (Sigma) was dissolved in corn oil (Sigma) at a concentration of 20 mg/ mL, and Cx3cr1CreER mice were administered the mixture (9 mg/40 g body weight; i.p.) every other day 5 times starting at 2–3 months old. After 2 days, the mice received unilateral injections to allow comparisons against the contralateral side, with AAV‐control or AAV‐NgR. The pAAV‐CMV‐DIO‐NgR‐GFP (AAV‐NgR) and a AAV vector that expressed GFP alone (AAV‐Control) were purchased from BrainVTA (Wuhan). Mice were anesthetized with ketamine/xylazine (30 mg/kg, 4 mg/kg) and immobilized using a stereotactic device. Before injections, AAV vectors were diluted with sterile PBS (pH 7.4) to achieve a titer of 2 × 10^11^. Viral preparations in 2‐μL volume were injected unilaterally into the hippocampus (anteroposterior –3.2, lateral 2.7 at 2.5 mm relative to the bregma). The preparation was injected with a speed of 0.5 μl/min over a period of 4 min by using a Hamilton 5‐μL syringe and a 27 G needle. 21 days after AAV injection, mice were received intra‐hippocampal injection of fibrillar Aβ. A 0.5‐mm burr hole was drilled in the skull, and 1 μl of fibrillar Aβ1‐42 (2 μg/μl) with Nogo‐p4/Rtn‐p4 (5 μmol/μl) solution injected intra‐hippocampal into the right hemisphere at a rate of 1 μl/min using a 5‐μl Hamilton syringe. Mice were sacrificed 2 days after injection and prepared for vivo Aβ phagocytosis.

#### 4.9.7 Microglial *in vivo* phagocytosis of methoxy‐X04–labeled Aβ

*In vivo* Aβ phagocytosis was determined using mice intraperitoneally injected with 10 mg/kg methoxy‐X04 (50% DMSO/50% NaCl [0.9%], pH 12) 3 h before sacrifice. Mice were perfused with ice‐cold PBS, and the brains were removed, minced using scalpels, and incubated in HBSS and 10% FCS containing 0.144 mg/ml collagenase type IV (Sigma, Aldrich) for 1 h at 37°C. Homogenization was achieved by pipetting through a 19‐gauge needle. The homogenate was filtered through a cell strainer (70 μm) and centrifuged at 155 g at RT for 10 min without using the brake. The pellet was resuspended in 37% percoll in PBS, underlayered with 70% percoll in PBS, and overlayered with ice‐cold PBS. The gradient was centrifuged at 800 g at 4°C for 25 min without using the brake. Microglial cells were recovered from the 37%/70% percoll interphase, diluted with 3 vol PBS, and centrifuged at 880 g at 4°C for 25 min without a break. The pellet containing the microglial cells was resuspended in 200 μl PBS. For flow cytometry analysis, 50μl of cells was diluted with 0.5 ml HBSS and centrifuged at 250 g for 5 minutes at 4°C. Binding of antibodies to Fc receptors was prevented by adding 1 μg of Fc block (BD Bioscience). Cells were taken up in 50 μl of primary antibody mix (CD11b‐APC, 1:100, BioLegend and CD45‐FITC, 1:100, eBioscience) and incubated for 30 min on ice. Cells were centrifuged at 250 g for 5 min at 4°C and resuspended in 200 μl HBSS. Corresponding isotype control antibodies were used for control and compensation. Cells were measured on a FACSCanto II Flow Cytometer (BD Bioscience), and data were analyzed using the flow cytometry software FlowJo (TreeStar). For analysis, the CD11b+CD45‐ population was gated. WT mice injected with methoxy‐X04 were used to determine the methoxy‐X04 threshold for non‐phagocytosing cells, and unstained WT cells were used to determine background fluorescence.

#### 4.9.8 Statistical analysis

Data shown are mean ±standard error of the mean (SEM) of at least three independent experiments. The differences were considered to be significant at *p* < 0.05. Statistical comparisons were made with GraphPad Prism 7.0 software using 2‐tailed Student's *t*‐test (for 2 groups meeting normal distribution criteria) or 2‐way ANOVA with Bonferroni's multiple‐comparison post hoc test (for groups across 2 variables, with multiple comparisons between groups). The behavioral experiments as summarized in Figure 2 were statistically analyzed using 2‐tailed Student's *t*‐test (NOR) and 2‐way ANOVA with Bonferroni's multiple‐comparison post hoc test (MWM). Where statistical significance is evaluated, variance between groups is confirmed to be similar between comparison groups and the statistical analysis is deemed appropriate. For data sets with sufficient n to analyze population distribution, tests for normality were administered (Anderson–Darling, D’Agostino & Pearson, Shapiro–Wilk, Kolmogorov–Smirnov).

## CONFLICT OF INTEREST

The authors declare no financial or commercial conflict of interest.

## AUTHOR CONTRIBUTIONS

HL, JW, and XQ conceived and participated in the design of the study. JW, XQ, and MH mainly wrote the manuscript, performed experiments, and confirmed all data analyses in all figures and supporting information. HS and QL participated in preliminary manuscript preparation and experiments. JW, QL, CG, and XH performed molecular/cellular and *in vivo* experiments. All authors read and approved the final manuscript.

## Supporting information

Fig S1

Fig S2

Fig S3

Fig S4

## Data Availability

All data generated and/or analyzed during this study are included in this article, and the data that support the results of this study are available from the corresponding author upon reasonable request. The English in this document has been checked by at least two professional editors, both native speakers of English. For a certificate, please see: http://www.textcheck.com/certificate/TIG3Ou
