# Supplementary figures and images for: Nogo receptor impairs the clearance of fibril amyloid‐β by microglia and accelerates Alzheimer’s‐like disease progression
Source: Aging Cell. 2021 Nov 24;20(12):e13515. doi: 10.1111/acel.13515 (PMC8672787; doi:10.1111/acel.13515)

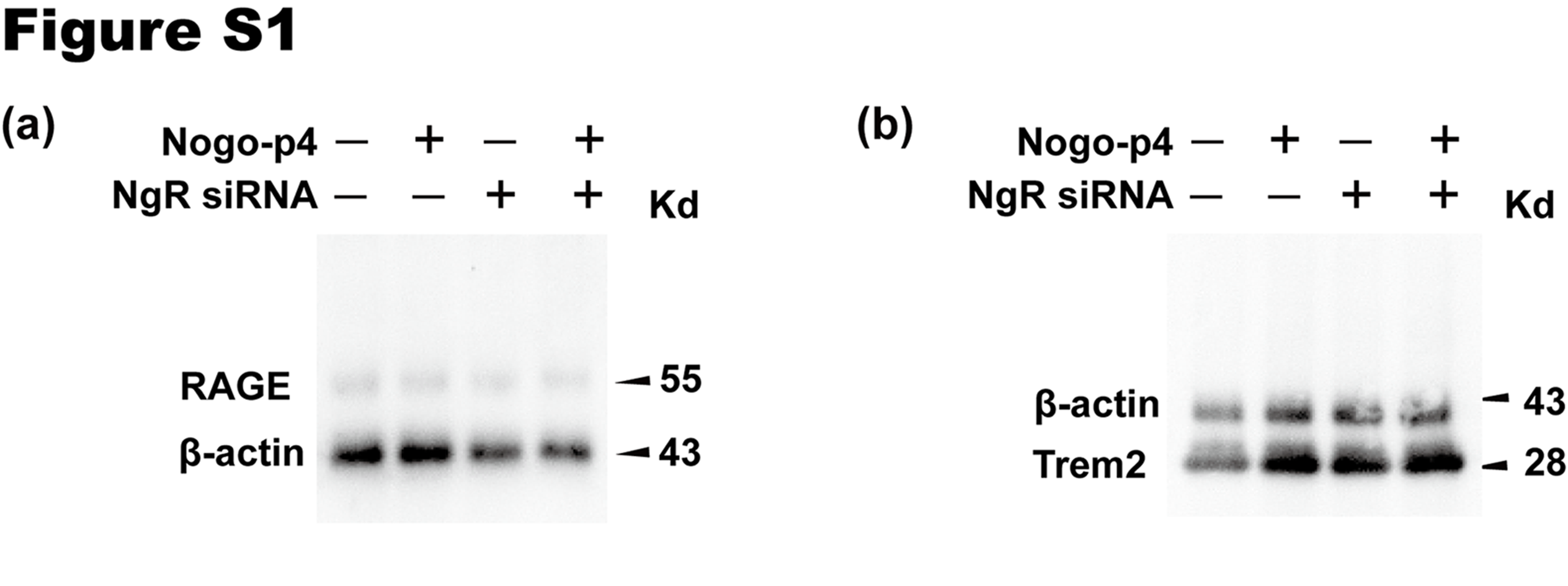

Supplement: Supplementary file 1 — Fig S1 [file ACEL-20-e13515-s003.png]

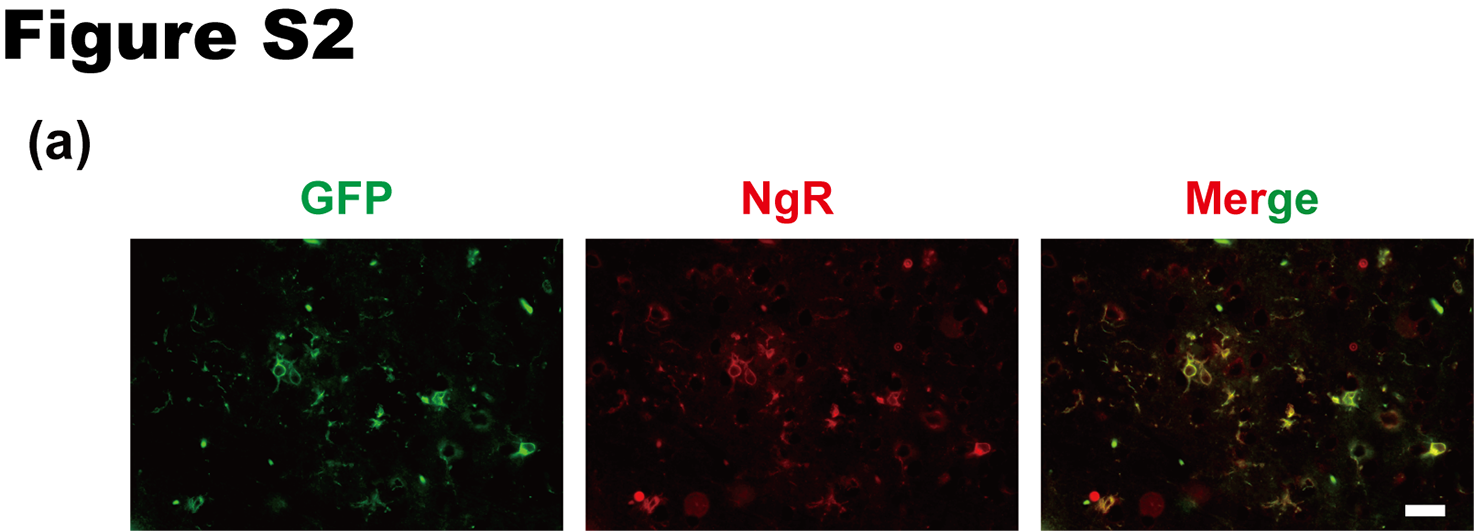

Supplement: Supplementary file 2 — Fig S2 [file ACEL-20-e13515-s004.png]

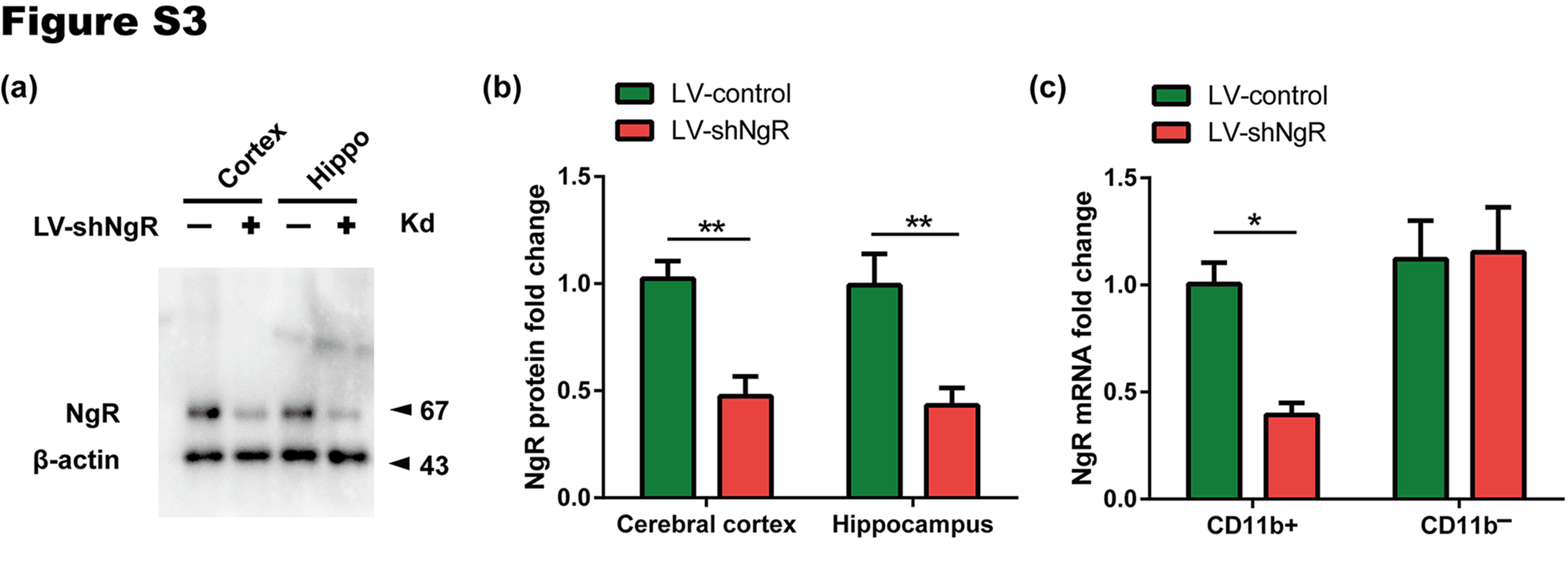

Supplement: Supplementary file 3 — Fig S3 [file ACEL-20-e13515-s002.png]

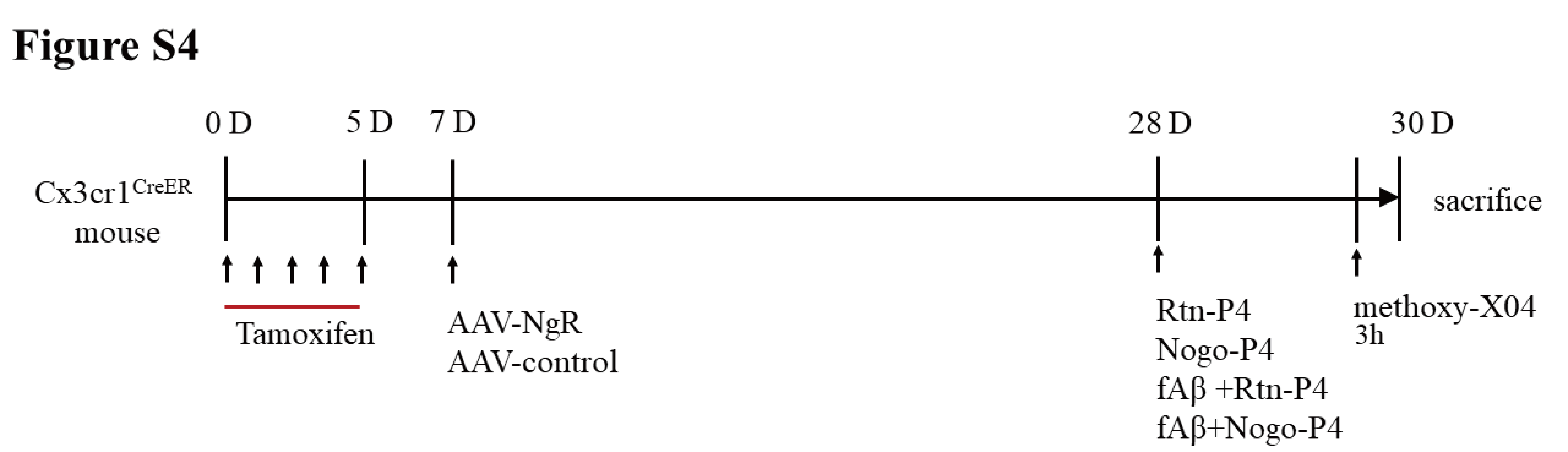

Supplement: Supplementary file 4 — Fig S4 [file ACEL-20-e13515-s001.png]
